# P13 Evaluation of *in vitro* activity of double β-lactam ‘therapy’ in ESBL+/CPE+ *Escherichia coli*—what is the association between ‘synergy’ and *in vitro* susceptibility?

**DOI:** 10.1093/jacamr/dlad143.017

**Published:** 2024-01-03

**Authors:** J Suich, M Van Der Woude, D Wearmouth, P Burns, G Barlow

**Affiliations:** Hull Teaching Hospitals, Hull, UK; York University, York, UK; York University, York, UK; Hull Teaching Hospitals, Hull, UK; Hull Teaching Hospitals, Hull, UK; York University, York, UK

## Abstract

**Background:**

With the increasing emergence of widespread antimicrobial resistance (AMR), finding therapeutic options for MDR organisms (MDRO), carbapenem-resistant Enterobacterales (CRE) and ESBL producers continues to represent a significant challenge. A promising therapeutic strategy against AMR is combination therapy, with the potential for synergistic drug interactions, reduced drug toxicity through reduced dosage drug–drug additive effects, repurposing of existing drugs, and the hope of overcoming resistance mechanisms and limiting resistance development.

**Methods:**

We systematically determined the relationship between double β-lactam therapy (*N*=390 combinations) against five *Escherichia coli* strains of variable resistance *in vitro* (three clinical and two purchased isolates). This included ESBL producers (*N*=2) and carbapenemase producers (CPEs) (*N*=3). For each of 13 β-lactam antibiotics, the MIC was determined individually, and subsequently in combination, using the MTS^™^ ‘cross’ synergy method (Liofilchem, 2012). The FIC was calculated by dividing the MIC of each drug in combination by the MIC of each drug alone, and adding the results (Figure 1). Combinations were performed in duplicate, with a third combination performed if there was any discrepancy in result (∼18.7%). Individual and combination antibiotic results were next analysed according to EUCAST clinical breakpoints to determine if synergistic combinations correlated with a potentially improved *in vitro* susceptibility phenotype. A susceptibility phenotype improvement was defined: R/R or R/I or I/I monotherapy, improving to R/S, I/S or S/S combination therapy (where S=susceptible, I=intermediate and R=resistant).

**Results:**

Overall, 64/390 (16.4%) combinations showed synergy; 106/390 (27.2%) were additive; 220/390 (56.4%) were indifferent. No antagonism was identified. Synergy was most commonly detected in ESBL producers (49/64;76.6% of synergistic combinations) and less frequently in CPEs (15/64; 23.4% combinations) (Figure 2). Of the synergistic combinations, 56/64 had ≥1 β-lactamase inhibitor present (87.5%). A total of 137/780 (17.6%) duplicate combinations showed improvement in EUCAST susceptibility between monotherapy and combination therapy (i.e. ≥1 R individually becoming ≥1 I or S in combination, or ≥1 I individually becoming ≥1 S in combination). But only 29/780 (3.7%) resulted in an R/R, R/I or I/I phenotype as monotherapy becoming ≥1 S in combination. Of the 137 duplicate combinations that improved, 55 (40.1%) were synergistic, 48 (35.1%) were additive and 34 (24.8%) were indifferent (by FIC index). Of 643/780 duplicate combinations that showed no improvement in susceptibility, 73 (11.35%) were synergistic, 164 (25.2%) were additive and 406 (63.45%) were indifferent. Of note, in 131/780 (16.8%) initial monotherapy susceptibility was S/S (i.e. further improvement not possible). Of these results, 33 (25.2%) were synergistic, 51 (38.9%) were additive and 47 (35.9%) were indifferent. Synergy was more common in combinations that improved by EUCAST susceptibility versus those that did not (χ^2^, *P*=0.00001). Ceftazidime/avibactam was the antibiotic most commonly present in synergistic combinations (27/64; 42.2%). Ceftazidime/avibactam/aztreonam was the combination most commonly associated with synergy (5/5 isolates) (Figure 3).

**Conclusions:**

Synergy was most commonly detected in ESBL producers with most synergistic combinations containing one or two β-lactamase inhibitors. Synergy was less commonly seen in CPE producers. Overall, improvement in *in vitro* susceptibility by EUCAST criteria was infrequent, and is highly bug–drug–drug combination dependent, but there is a statistically significant association between EUCAST susceptibility improvement and synergistic combinations. Further work using alternative validating methodology and in more isolates is required.

**Figure 1.**
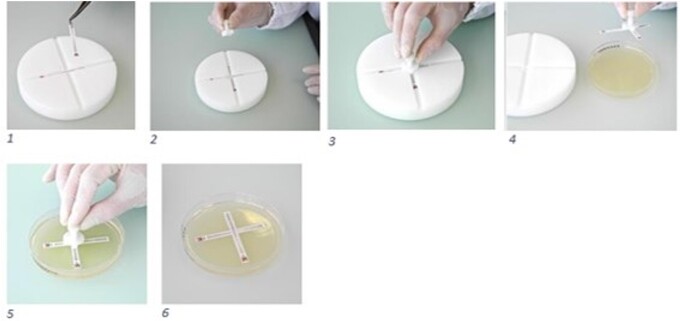
MTS™ ‘cross’ synergy method (Liofilchem, 2012). FIC index calculations were used to interpret findings, whereby FIC = (MICA combination A+B/MIC agent A) + (MICB combination A+B/MIC agent B). A result of ≤0.5 was taken to indicate ‘synergy’, >0.5 and ≤1.0 to indicate ‘additive’ effect, >1.0 and ≤4.0 to indicate ‘indifference’ and >4.0 to indicate ‘antagonism’.

**Figure 2.**
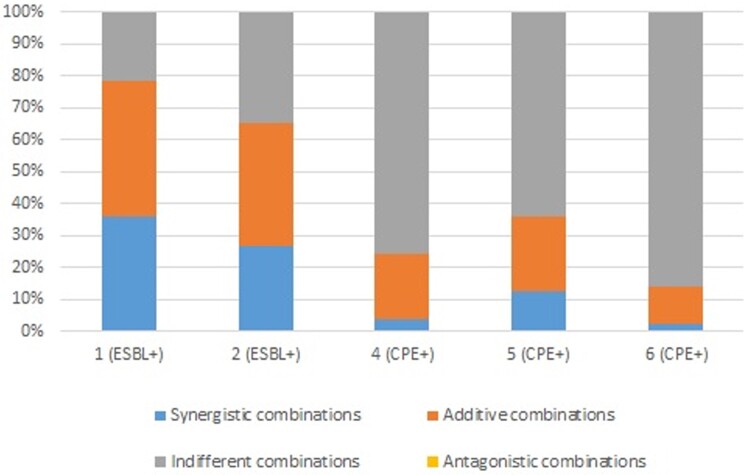
β-Lactam antibiotics in combination versus five *E. coli* isolates of variable resistance *in vitro*.

**Figure 3.**
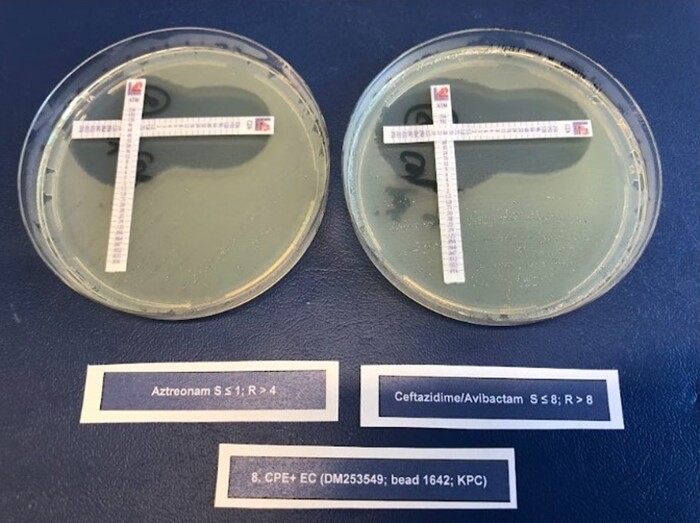
Ceftazidime/avibactam/aztreonam was the combination most commonly associated with synergy.

